# Innovative Technical Solution Using the Renal Artery Stump after Nephrectomy as an Inflow Artery for Lower Limb Revascularization—A Case Report

**DOI:** 10.3389/fsurg.2022.864846

**Published:** 2022-05-13

**Authors:** Eliza Russu, Adrian Vasile Mureșan, Reka Kaller, Lucian Toma, Cătălin Mircea Coșarcă, Călin Bogdan Chibelean, Emil Marian Arbănași, Eliza Mihaela Arbănași

**Affiliations:** ^1^ Clinic of Vascular Surgery, Mures County Emergency Hospital, Targu Mures, Romania; ^2^ Department of Surgery, George Emil Palade University of Medicine, Pharmacy, Science, and Technology of Targu Mures, Targu Mures, Romania; ^3^ Clinic of Urology, Targu Mures County Hospital, Targu Mures Romania; ^4^ Faculty of Pharmacy, George Emil Palade University of Medicine, Pharmacy, Science, and Technology of Targu Mures, Targu Mures, Romania

**Keywords:** aortic aneurysm, critical limb ischemia, renal artery, nephrectomy, lower limb revascularization, vascular surgery

## Abstract

We present the case of a 56-year-old patient admitted to the vascular unit of the Targu Mures County Emergency Clinical Hospital after a computed tomography angiography performed for critical limb ischemia showed a tumor of the right kidney of 11.3/12/11 cm anteroposterior/later-lateral/craniocaudal, accompanied by an abdominal aortic aneurysm (AAA) (3 cm diameter) and right iliac artery occlusion. An interdisciplinary team formed of urological and vascular surgeons decided and performed a one-step operation. The right kidney was removed, and the limb revascularization was achieved by performing a bypass that used the right renal arterial stump as an inflow artery, thus called a reno-femoral bypass. The AAA had no indication for reconstruction. The final pathology interpretation of the kidney tumor revealed a clear cell renal cell carcinoma, excised with oncological safety margins. A short-term follow-up found the patient without ischemic symptomatology and a fully functional graft.

## Introduction

Peripheral artery disease (PAD) morbidity has seen a significant increase in the last 20 years, currently affecting 1,466 people out of 100,000 worldwide, compared with 1,229 out of 100,000 in 2010. The countries with the highest incidence are Denmark, with 5,330 people out of 100,000, and the United States, with 3,799 people out of 100,000 (1).

Chronic limb-threatening ischemia (CTLI) is a severe stage of PAD and corresponds to stages III–IV Leriche-Fontaine (2), or 4–6 Rutherford (3), and is associated with a high rate of amputation and mortality (4, 5). CTLI is characterized by rest pain, tissue damage, or tissue necrosis (6, 7). Striving for endovascular or surgical revascularization is mandatory to preserve the viability and function of the affected limb (6).

PAD’s concomitance with malignancy is well known and heavily studied. The disease has the same risk factors, such as smoking, obesity, and diabetes, and some similarities regarding the physiopathological mechanisms (8). In a manuscript published by El Sakka et al., the authors could signal malignancy in 22 (11.5%) out of the 192 studied critical limb ischemia patients (9). Also worth citing is the paper of Nicolajsen et al., which reported 1 out of 5 acute limb ischemia patients having a malignancy (10).

In terms of incidence rate, kidney tumors occupy the fourteenth place worldwide as newly diagnosed cases in 2020, accounting for 431,288 patients. They are in the fifteenth place regarding mortality rates, accounting for 179,368 fatalities (11).

Abdominal aortic aneurysm (AAA) and malignancy concomitance were also studied in detail, casting a shadow on the dispute over the one-stage vs. two-stage therapeutical approach (12). The 2019 European Society for Vascular Surgery Clinical Practice Guidelines on the Management of Abdominal Aorto-iliac artery Aneurysms have a specific set of references about the issue of concomitant malignant disease. Still, these are recommendations for managing an aneurysm with a particular diameter (over 5.5 cm) (13). AAA has a high mortality rate and may evolve toward retroperitoneal rupture, aorto-enteric fistula formation, or spontaneous thrombosis (14, 15).

This paper aims to present an innovative surgical technique of using the remaining renal artery, after nephrectomy, as an inflow artery for the revascularization of the critical ischemic leg, in a patient presenting with an infrarenal AAA and the occlusion of the common and external right iliac artery.

## Case Presentation

**Figure 1:**
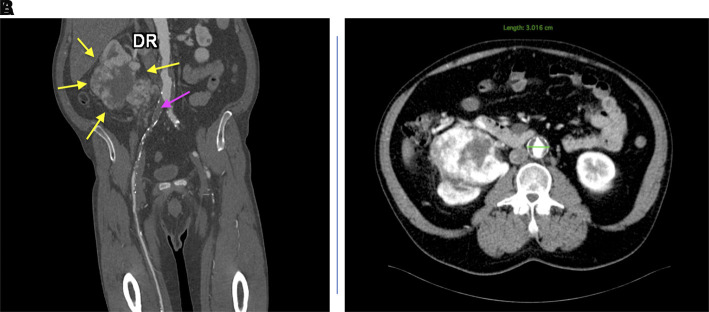
Computed tomography angiography before surgery: coronal section, yellow arrows: kidney tumor; purple arrow: occlusion of the right common and external iliac arteries (**A**) and axial section, AAA with intraluminal thrombus; renal tumoral mass with central necrosis (**B**).

A 56-year-old male patient presented to the vascular unit of the Targu Mures County Emergency Clinical Hospital complaining of severe pain in the right leg, without any relief after taking common analgesic drugs. The patient had no right femoral pulse, with the other clinical signs of an ischemic leg being the absence of hair growth on the right calf, right big toe recurrent mycosis, and a positive Buerger and Ratschow test. He had rest pain, and the measured ankle-branchial index (ABI) was 0.4. One month prior, he was referred to an angiologist, having had claudication in the right leg, and was advised toward smoking cessation and exercise rehabilitation, and was put on first-line medication (cilostazol), in addition to his chronic antihypertension drugs. The laboratory findings revealed slight anemia (Hgb 9.97 g/dl). Doppler examination and computed tomography (CT) angiography jointly diagnosed right common and external iliac artery occlusion type D transAtlantic inter-society consensus (TASC), an infrarenal AAA with a 3- cm diameter with intraluminal thrombus, and a right renal tumor of 11.3/12/11 cm (anteroposterior/later-lateral/craniocaudal), with central necrosis (Figure 1).

The patient also had hypertension and a body mass index of 30.67. Echocardiography showed an ejection fraction of 48% and mild hypokinesia of the lateral wall of the left ventricle. All the kidney function parameters were normal: blood urea nitrogen (BUN) and creatinine. He had an average glomerular filtration rate.

After a senior urologist analyzed the CT angiography, the kidney tumor presented high malignancy characteristics, and nephrectomy was elected as the procedure of choice. After discussing the case in a multidisciplinary meeting, a one-stage open repair was selected, forming an interdisciplinary team with the Urology Department. Endovascular repair (EVAR) was ruled out due to occlusion of the right common and external iliac arteries and atherosclerotic lesions affecting the controlateral iliofemoral axis. The aneurysmal neck was also right below the origin of the renal arteries, rendering the local situation anatomically hostile and leaving little space for clamping or a potential anastomosis. A lateral clamping would have engaged the aneurysmal wall and the intraluminal thrombus. A potential clamping above the renal arteries would have exposed the left (remaining) kidney to further ischemic injuries. Given a young and active patient with a reasonable life expectancy, the femoral–femoral crossover bypass was also ruled out, as it had the potential to harm the donor artery leg. A decision was then taken to leave a right renal artery stump of cca 3 cm, which would allow a safe clamping and a further “noli-me-tangere” of the aortic aneurysm, leaving it, in the future, to be possibly solved by an EVAR with a left femoral approach. After a complete exclusion of all the other scenarios, the decision to perform a reno-femoral bypass was made, considering that the right renal artery was not included in the tumoral mass. Senior vascular and urology surgeons were to perform the surgical procedure in the Vascular Unit Operating Room within the Târgu-Mureș County Emergency Clinical Hospital. The patient was also inclined to favor the one-stage surgery after presenting the options.

**Figure 2:**
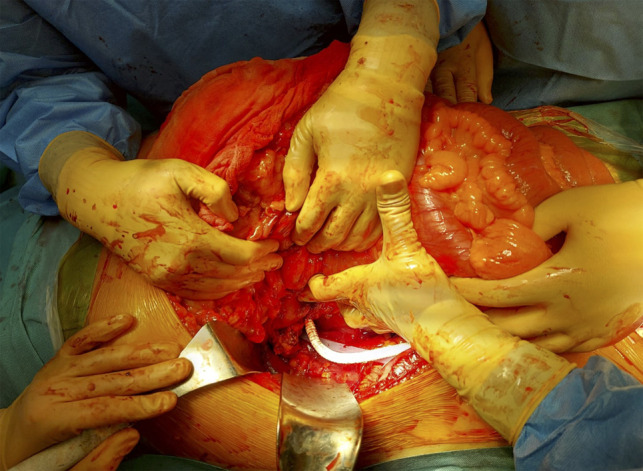
Proximal anastomosis (intraoperative photo).

Using a Jalaguier modified incision, the right kidney was first removed, guided by oncological safety principles, after a biopsy was collected and sent to the morphopathology compartment. The result showed malignancy. The whole procedure took place in a retroperitoneal manner. The vascular team distally prepared, clamped, and heparinized the right common femoral artery. A right renal artery stump of 3 cm was clamped safely, leaving enough space for an end-to-end anastomosis to an externally ringed polytetrafluoroethylene graft (7- mm diameter), performed using a continuous suture with Surgipro™ Monofilament Polypropylene 5-0. The team chose an externally ringed graft for safety reasons, as predicting an evolution free from a local tumoral recurrence was problematic, although perirenal fat was excised and radical lymphadenectomy performed. In this scenario, the best-performing graft against external compression had to be the reasonable option. The graft was then tunneled retroperitoneally in a straight, non-kinked position, taking care of the angles of the proximal anastomosis (Figure 2).

The distal anastomosis was performed end-to-side to the common femoral artery, using a continuous suture with Surgipro™ Monofilament Polypropylene 5-0. The back-bleeding from the profunda femoris artery was vigorous, and no thromboendarterectomy of the common femoral artery was needed.

Early postoperatively, anticoagulation was achieved by administering heparin intravenously, 2,500 IU (international units) every 3 h. We monitored the activated partial thromboplastin time values.

The results were satisfactory postoperatively: palpable femoral, popliteal, distal pulses, and an ABI of 0.9. Ultrasound examination showed triphasic Doppler waveforms from the common femoral artery to the ankle. The patient was discharged without any complications on the 7th postoperatory day, with the anemia corrected and the graft patent. Oral anticoagulants, coumarin-like, were prescribed, as well as clopidogrel. BUN and creatinine were elevated postoperatively, but they began to decrease. The patient had a creatinine value of 3.15 mg/dL at discharge. He did not require a urinary catheter. He was instructed to monitor BUN and creatinine values and the Index Normalized Ratio to explore the coagulation status and was referred to a nephrology specialist.

The final pathology result was Clear Cell Renal Cell Carcinoma of the right kidney, International Society of Urological Pathology grade 2, Fuhrman grade 2, infiltrating the perirenal fat, tumor stage: pT3a. The margins of the excision–renal artery section were negative. The patient was referred to an oncologist and started chemotherapy.

**Figure 3:**
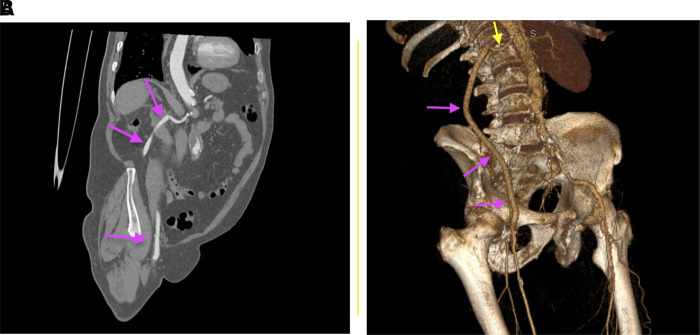
CT angiographic after surgery: coronal section, purple arrows: reno-femoral permeable bypass (**A**) and 3D reconstruction, yellow arrow: right renal artery; purple arrows: reno-femoral permeable bypass (**B**).

One-month follow-up found a patent graft, palpable pedal pulses, a lack of symptomatology, and an ABI of 0.9. Three-month postop control CT angiography showed a fully functional graft (Figure 3).

## Discussion

Tumoral active status patients have a high risk of thromboembolic complications, with an up to 70% increase of the occurred episodes up to 5 months before the oncological diagnostic. The percentages remain high in the first 6 months after (16–18).

The staging of the renal cell carcinoma (RCC), evaluated as thoroughly as it can be done before having a biopsy performed, is mandatory. Also, having accidentally found a kidney tumor in a young patient with a reasonable life expectancy, time is crucial. The American Cancer Society reported, in 2018, a 5-year survival rate for RCC stage 2 of 74%, thus establishing RCC treatment as a priority. Still, limb revascularization in a CTLI patient remains an against-the-clock issue.

The synchronous approach for AAA and nonvascular procedures was demonstrated to be safe by Ochsner et al., with low rates of allograft infection if done in a manner of first implanting the graft, carefully closing the retroperitoneum over it, and then managing the nonvascular procedure (19). The recommendation was deemed reasonable by several studies and reviews, such as those of Prusa and Mohandas, as staging the procedure would only lead to complications due to scar tissues and even more difficult anatomy (20). AAA associated with RCC was described in several papers, emphasizing the good outcomes after open aortic repair, being done simultaneously with nephrectomy procedures (21).

Nephron-sparing operations were postulated as the standard of choice, as prolonged clamping time is complication-inducing (22). Intra-operative findings guide the surgeon toward the best clamping site after analyzing the CT angiography 3D reconstruction. In a patient undergoing a nephrectomy, clamping above the renal arteries is not desirable. If below the renal arteries origin, the aortic wall is not suitable, as in highly calcified or aneurysmal, a technical challenge arises, as was our case.

The concomitance of an AAA, a tumor, and diffuse atherosclerotic lesions forming a TASC D pattern affecting the aortoiliac configuration with minimal infrainguinal damage creates a challenge in finding an optimal revascularization solution. As the AAA has no indication for repair due to its diameter, a revascularization solution taken into discussion could be an extraanatomical bypass. Given that the left femoropopliteal axis is also affected by atherosclerotic lesions, performing a femoral–femoral crossover bypass or even a left common iliac to right femoral bypass may lead to the left leg claudication onset, precipitated by the hemodynamic steal syndrome. The axillofemoral extra anatomical bypass could have been the procedure of choice for a two-stage surgery: nephrectomy followed by the bypass, with the same objection remaining—an extraanatomical graft with non-optimal patency in a young, active patient with a reasonable life expectancy given by the radical nephrectomy.

The choice of not touching the AAA and using the renal artery stump makes way for a different therapeutical solution to be contemplated, should the need emerge for a future EVAR procedure with a left femoral approach.

Given a 56-year-old patient who preferred a one-stage operation, the need for radicality in the oncological setting, and the extended atherosclerotic lesions of the iliac artery, forming an interdisciplinary team and using a technical innovation were the best choices to strive for the best solution. As far as we know, this type of revascularization has not been reported previously.

## Conclusion

As specific situations require specific solutions, the choice of using the renal stump artery was, given the case at hand, the only logical one. Considering a technical innovation is, in complex cases, better than risking inflicting further complications due to prolonged clamping time.

## Data Availability

The original contributions presented in the study are included in the article/supplementary material; further inquiries can be directed to the corresponding author/s.
